# Usutu Virus Isolated from Rodents in Senegal

**DOI:** 10.3390/v11020181

**Published:** 2019-02-21

**Authors:** Moussa Moïse Diagne, Marie Henriette Dior Ndione, Nicholas Di Paola, Gamou Fall, André Pouwedeou Bedekelabou, Pape Mbacké Sembène, Ousmane Faye, Paolo Marinho de Andrade Zanotto, Amadou Alpha Sall

**Affiliations:** 1 Arboviruses and Hemorrhagic Fever Viruses Unit, Virology Department, Institut Pasteur de Dakar, BP220 Dakar, Senegal; Marie.NDIONE@pasteur.sn (M.H.D.N.); gamou.fall@pasteur.sn (G.F.); ousmane.faye@pasteur.sn (O.F.); Amadou.SALL@pasteur.sn (A.A.S.); 2 Department of Animal Biology, Faculty of Science et Technics, Université Cheikh Anta Diop de Dakar (UCAD), BP 5005 Fann, Dakar, Senegal; mbacke.sembene@ucad.edu.sn; 3 Laboratory of Molecular Evolution and Bioinformatics, Department of Microbiology, Biomedical Sciences Institute, University of Sao Paulo, Sao Paulo 05508-000, SP, Brazil; nicholasdipaola@gmail.com (N.D.P.); pzanotto@usp.br (P.M.d.A.Z.); 4 Center for Genome Sciences, United States Army Medical Research Institute of Infectious Diseases (USAMRIID), Fort Detrick, Frederick, MD 21702, USA; 5 Ecole Inter-Etats des Sciences et Médecine Vétérinaires de Dakar, BP5077 Dakar, Senegal; pouwedeouandre@gmail.com; 6 BIOPASS (IRD-CBGP, ISRA, UCAD), Campus de Bel-Air, BP 1386, CP 18524 Dakar, Senegal

**Keywords:** Usutu virus, arbovirus, Senegal, rodents, in vivo experiment

## Abstract

Usutu virus (USUV) is a *Culex*-associated mosquito-borne flavivirus of the Flaviviridae family. Since its discovery in 1959, the virus has been isolated from birds, arthropods and humans in Europe and Africa. An increasing number of Usutu virus infections in humans with neurological presentations have been reported. Recently, the virus has been detected in bats and horses, which deviates from the currently proposed enzootic cycle of USUV involving several different avian and mosquito species. Despite this increasing number of viral detections in different mammalian hosts, the existence of a non-avian reservoir remains unresolved. In Kedougou, a tropical region in the southeast corner of Senegal, Usutu virus was detected, isolated and sequenced from five asymptomatic small mammals: Two different rodent species and a single species of shrew. Additional molecular characterization and in vivo growth dynamics showed that these rodents/shrew-derived viruses are closely related to the reference strain (accession number: AF013412) and are as pathogenic as other characterized strains associated with neurological invasions in human. This is the first evidence of Usutu virus isolation from rodents or shrews. Our findings emphasize the need to consider a closer monitoring of terrestrial small mammals in future active surveillance, public health, and epidemiological efforts in response to USUV in both Africa and Europe.

## 1. Introduction

In 1959, the Usutu virus (USUV) was first isolated in South Africa [1]. Part of the genus *Flavivirus* and the Flaviviridae family, USUV is a mosquito-borne agent capable of causing symptomatic infections in birds and humans [2]. Although subsequent infections in sub-Saharan Africa were detected in ornithophilic mosquito species [3,4], the threat of USUV was heightened in 2001 when it was detected in Vienna, Austria [5]. Active surveillance began in both retrospective and incoming serological samples from birds and mosquitoes. As a result, USUV was detected in bird samples from 1996 localized in Tuscany, Italy, marking the earliest known presence of USUV in Europe [6].

In the subsequent years, USUV incidence has increased as it has spread across Europe [7]. Like West Nile virus (WNV) and other species within the *Culex*-associated mosquito-borne flaviviruses group, USUV can infect and replicate in a large number of mosquito and bird species, which act as vectors and amplifying hosts, respectively [8,9,10]. Recent evidence suggests that bats could also be a reservoir for USUV [11]. Immunosuppressed patients exhibiting meningoencephalitis also have tested positive for USUV [12,13,14]. Responding to the growing concern for USUV and human health, the European Ministry of Health and several serological surveillance programs in Germany, Italy, and Croatia amassed and consequently revealed asymptomatic and a few symptomatic infections in healthy blood donors [15,16,17]. Within the Flaviviridae family, several tick-borne flaviviruses (TBFV) utilize rodents and other small mammals in their maintenance cycle. Tick-borne encephalitis virus, Omsk hemorrhagic fever virus, Kyasanur Forest virus, Apoi virus, and Powassan encephalitis virus are well-studied examples [18,19,20,21,22]. However, there have been isolations of flaviviruses that suggest the existence of transmission cycles involving rodents outside of the TBFV clade; one example being the isolation of Koutango virus from *Tatera kempi* (Kemp’s gerbil) in 1968 [23]. More recently, a strain of Wesselsbron virus was isolated from rodents in the southeastern part of Senegal [24].

Here, we present five complete viral genomes of USUV strains isolated from two different rodent species and one shrew species from Kedougou, Senegal. At the time of capture, the animals did not show signs of disease, suggesting a possible new transmission vector or reservoir for USUV. To our knowledge, this is the first report of USUV isolation from rodents and shrew. Our findings could play a role in future efforts regarding the surveillance, public health, and epidemiology of USUV in Africa and Europe.

## 2. Material and Methods

### 2.1. Study Site

Between May 2012 and December 2013, 32 small mammal trapping sessions were undertaken to study the circulation of arborviruses in some localities of Eastern Senegal (Table S1). Briefly stated, the trap was a locally made single capture wire-mesh live traps (8.5 × 8.5 × 26.5 cm) and Sherman folding box traps (8 × 9 × 23 cm) were both set inside dwelling places and their surroundings for sessions of one to six consecutive days, with peanut butter as bait [25]. One of each type of trap was set per room for the whole night and inspections were carried out each morning. Each trapped specimen was identified to the genus or species level based on morphological or geographical criteria [26,27] or, in case of ambiguity, by further molecular or chromosomal analyses [28,29,30]. After health observations, trapped individuals were euthanatized by cervical dislocation as previously recommended [31]. The brain, the tissues and the serum of each individual were separated and put in dry ice for transportation to Institut Pasteur de Dakar (IPD) and stored at −80 °C until further processing. The permission to work within the different villages was obtained from appropriate authorities, and animals were correctly treated, following the approved guidelines [32].

### 2.2. Arbovirus Screening and Isolation Attempt

Each sample was triturated in Leibovitz-15 (L-15) medium (GibcoBRL, Grand Island, NY, USA) containing penicillin and streptomycin (Sigma, GmBh, Mannheim, Germany) and 10% FBS (GibcoBRL, Grand Island, NY, USA), and centrifuged in order to collect the suspension.

RNA was extracted from these different suspensions using the QIAamp RNA Viral Kit (Qiagen, Hilden, Germany) according to the manufacturer’s recommendations and stored at −80 °C until use. A screening of these rodent samples was performed using conventional reverse transcription (RT)-PCRs, in order to target different arbovirus genera (Table S2). Virus isolation attempts were also performed from 150 µL of sample suspensions of each specimen that were inoculated separately onto monolayers *Aedes albopictus* C6/36 cells in 25-cm^2^ tissue-culture flasks. After an incubation step at 28 °C for a maximum of 7 days, the supernatant was collected and an aliquot of 200 μL was used for a new blind passage until passage 4 or after the observation of a cytopathic effect (CPE). The concerned supernatants were also tested for several arboviruses by conventional RT-PCRs.

### 2.3. RT-PCR and Sequencing of USUV Isolates

Genome sequences were obtained by overlapping RT-PCRs based on published USUV primers [33]. A second step of RT-PCRs was done with specific primers to obtain the complete genome. cDNA synthesis was done using an AMV reverse transcription kit (Promega, Madison, WI, USA) according to the manufacturer’s instructions, and PCR reactions were performed using Go-Taq PCR kit (Promega, Madison, WI, USA).

### 2.4. Phylogenetic Tree

Twenty-three complete polyprotein sequences of Usutu genomes with country and year of isolation were downloaded from Genbank for this study (Table S3). Sequences were labeled with relevant information and separated by an underscore: accession number, strain or isolate name, isolation source, country, and year of collection. With the addition of 5 new sequences obtained during this study, a total of 28 sequences were aligned using Muscle v3.8.31 [34] and manually curated using Se-Al v2 [35] and Geneious v9 [36]. For each alignment, we performed a recombination screening (RDP, GeneConv, Chimaera, MaxChi, BootScan and SiScan) in RDP4.61 [37]. A maximum likelihood tree was inferred with FastTree v2.1 [38] using a GTR+I nucleotide substitution model, pseudocounts, and the exhaustive search parameter. Local support values were calculated using the Shimodaira-Hasegawa (SH)test for 5000 bootstrap replications.

### 2.5. Virus Strains for In Vivo Experiments

The virus strains used in this study were the SAAR-1776 reference strain obtained from a mosquito, the HB81P08 strain isolated from a human with a neurological disease and an isolate obtained during this work. The virus stocks were prepared by inoculating C6/36 continuous cell lines for 4 days. The infection status was tested by immunofluorescence assay (IFA), real-time RT-PCR (Reverse Transcriptase-Polymerase Chain Reaction) and plaque assay as previously described [39]. The supernatant of infected cells was aliquoted, frozen at −80 °C, and used as viral stocks for animal experiments.

### 2.6. Animal Experiments

The effect of USUV infection on mouse growth and survival were studied by experiments performed with three different isolates, passaged 3 times in C6/36 cells. We used Swiss Webster (CFW) mice, produced and reared at the IPD farm, accredited by the WHO Collaborating Centre for Arboviruses and/or Hemorrhagic Fever Reference and Research. A viral dose of 10^3^ plaque forming units (pfu) of each strain in 0.1 mL phosphate buffered saline (PBS, pH 7.5) was inoculated onto a group of ten 3-to-4-week-old mice. Inoculations were done via 3 different routes: intracerebral (IC), intraperitoneal (IP) and subcutaneous (SC) injections. Ten control mice were included for the experiment. In order to reduce aggressiveness, we split each cohort into two groups of 5 individuals. Mice were identified using an "ear marking code". The weight was chosen as the growth indicator. Animals were monitored daily and were weighed using a spring balance with 1 g precision, every 2 days or after the observation of particular events (death, beginning of symptoms, etc.) until day 20 post infection (pi). All statistical inferences were calculated using the Wilcoxon rank sum test. Blood samples were taken just before death or during the last day of observation.

## 3. Results

### 3.1. Usutu Virus Detection in Rodents and Shrew

We trapped 1414 small mammals from nine different species in eastern Senegal during 32 trapping sessions (Table S1). Research of arboviruses in small mammal samples was realized by both molecular screening and isolation attempts on *Aedes albopictus* C6/36 cells. Amplification of partial polymerase gene by pan-flavivirus RT-PCR [16] on five supernatants of cytopathic effect-exhibiting C6/36, permitted the detection of a virus sharing 100% homology with the Usutu virus (USUV) prototype strain SAAR-1776 (Accession number: AF013412). These results were confirmed by using a USUV-specific qRT-PCR [40] on the different original brain homogenates samples. USUV-infected mammals belong to three different species of rodents and shrew, as seen in Table 1: One specimen of black rat, *Rattus rattus* (ROD259552); three multimammate mouse, *Mastomys natalensis* (ROD259466, ROD259496 and ROD259524); and one specimen of shrew, *Crocidura* sp. (CROC259520). USUV was the only virus found in these different samples.

**Figure 1:**
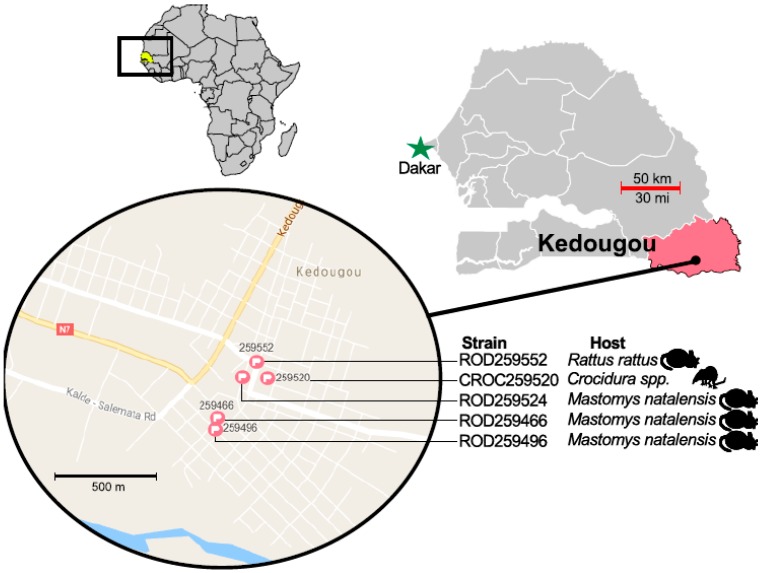
A map of Kedougou with the locations of the five captured Usutu virus-positive mammals.

They were all trapped in five neighboring constructions in Kedougou, as seen in Figure 1. No disease manifestations or symptoms that are possibly due to an infection were observed in the virus carriers at the time of capture. Conventional RT-PCRs were performed to obtain the complete polyprotein sequence for each strain with published overlapping primers [33] and new designed ones. The Genbank accession numbers of obtained sequences are listed in Table 1.

### 3.2. Map and Genomic Analysis

The sequences of the new isolates were highly similar to one another, with 1% divergence for both nucleotide and protein sequences. Moreover, when compared to reference strains, such as the Vienna2001 strain (Accession number: AY453411) and the SAAR-1776 strain (Accession number: AF013412), rodent sequences were 96.9% and 99.6% similar at the nucleotide level, respectively.

**Figure 2:**
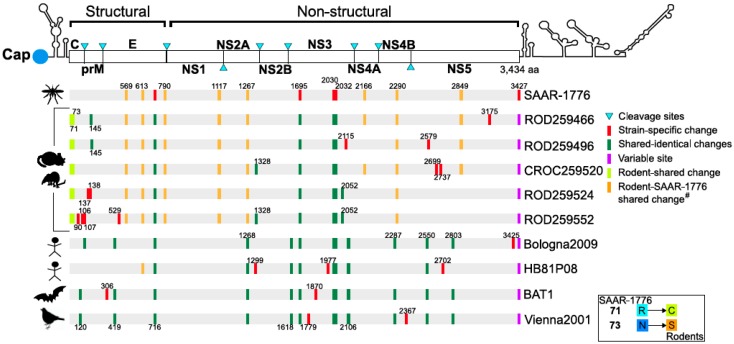
The comparative genome organization and mapping of amino acid differences between Usutu virus (USUV) sequences, characterized from different hosts. Positions are annotated and aligned to their loci in the USUV polyprotein. Host of origin and strain names can be found to the left and right of the polyprotein sequence maps, respectively. The shared non-synonymous changes from the SAAR-1776 to the rodent USUV strains are shown in the bottom right corner.

Using the SAAR-1776 sequence as a reference, we aligned and mapped amino acid differences across the entire polyprotein, as seen in Figure 2. We also added “hallmark” sequences of Usutu virus from different localities and hosts for a more robust comparison. Crucially, we found two identical substitutions present only in rodents and shrew sequences at 71st and 73rd codons in capsid (C) region (yellow markers). We then identified some substitutions shared only with the SAAR-1776 strain and rodent isolates (orange markers), positioned at codon sites: 569, 613, 790, 1117, 1267, 2166, 2290, and 2849. The unique and specific S613G mutation (envelope) is also shared with the human isolate from the Central African Republic (HB81P08, Accession number: KC754955). Furthermore, we discovered the substitutions that are unique for each rodent strain, which are marked red in Figure 2. SAAR-1776 specific amino acid differences were also observed in the envelope (E) protein (codon 716) and non-structural 3 (NS3) protein (codon 1695, codon 2030 and codon 2032).

### 3.3. Phylogenetic Analysis

**Figure 3:**
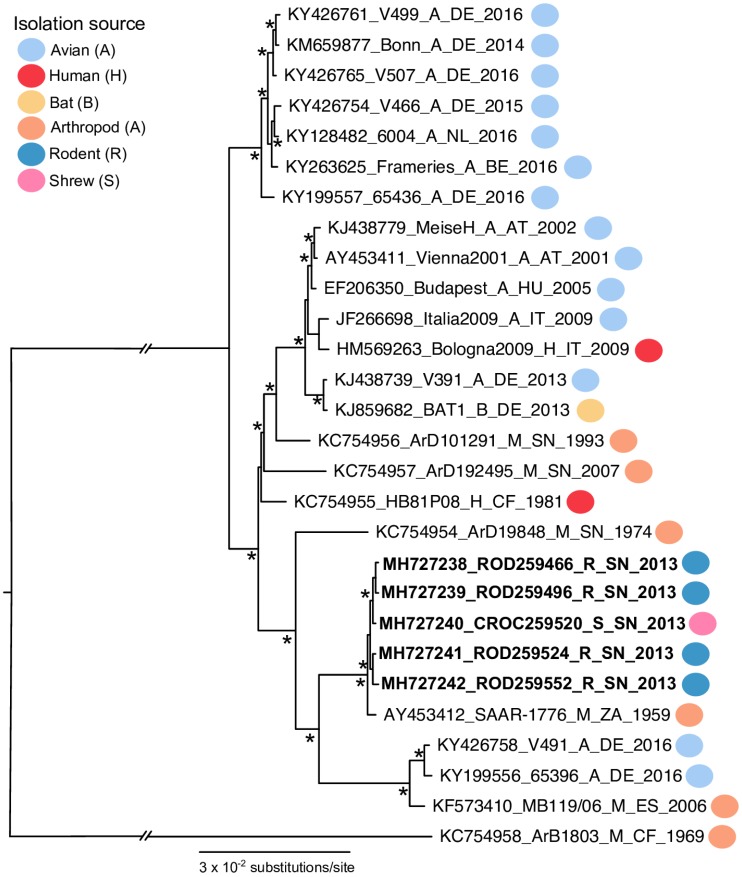
A maximum-likelihood phylogenetic tree estimated using 28 USUV polyprotein sequences. Rodent taxon labels are in bold. The colored circles indicate the host where the USUV sequence originated. The asterisks (*) at major nodes indicate an SH-like support value > 70%. The tree branches are scaled by the nucleotide substitutions per site.

A phylogenetic tree was used to infer the ancestry of the five USUV polyprotein sequences generated from rodents. The estimated maximum-likelihood phylogenetic tree had a similar topology to previous phylogenic estimations for USUV [41,42], as seen in Figure 3. All five rodent sequences were clustered within the Africa 2 genotype. Additionally, the rodent sequences shared a common ancestor with the SAAR-1776 sequence from South Africa. 

### 3.4. Comparative In Vivo Study of USUV Effect on Mouse Growth and Survival

**Figure 4:**
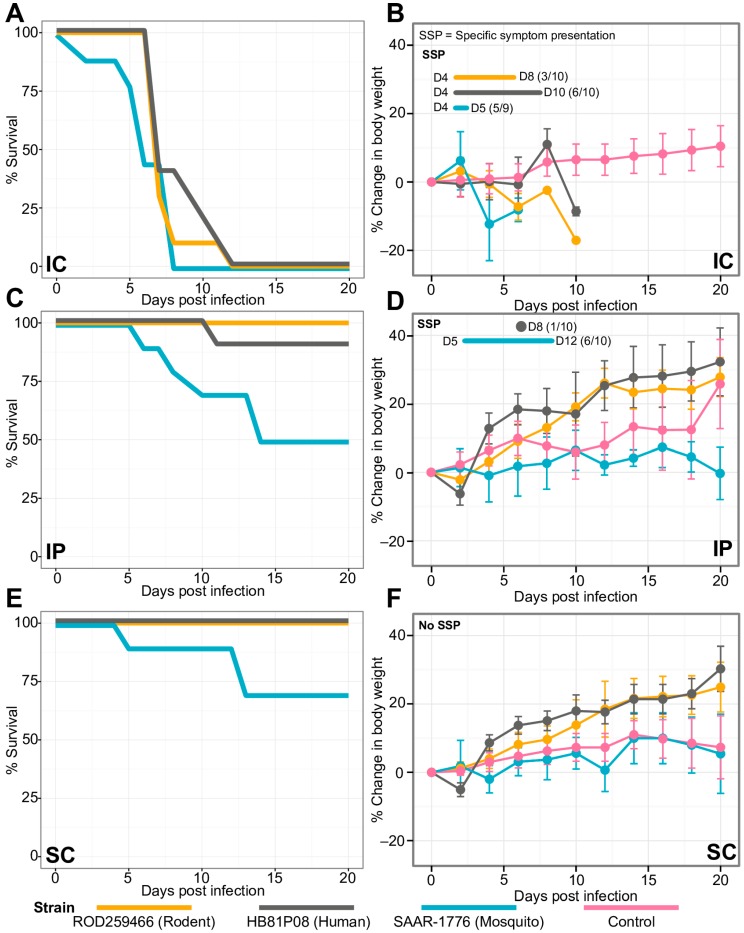
The in vivo evaluation of mouse mortality and virulence of three Usutu virus strains inoculated in 3-to-4-week-old Swiss mice. Survival (left panels) and changes in body weight (right panels) were observed after three different inoculation methods: intracerebral (IC, panels (**A**,**B**)), intraperitoneal (IP, panels (**C**,**D**)), and subcutaneous (SC, panels (**E**,**F**)) for 20 days, post-infection period. The control mice (red) were injected with PBS in duplicate groups using the same inoculation methods. Error bars show the 95% confidence intervals for each sampled time point. The days of disease-specific symptom presentations (SSP) is shown in the top left corner of panels (**B**,**D**,**F**). The number of days (**D**) post-infection when disease-specific symptoms are shown for each strain, as well as the proportion of symptomatic mice in each group.

To determine the USUV’s biological properties in the rodent isolate and its effect on mouse growth and development, it was compared using in vivo infection assays with (i) the SAAR-1776 reference strain obtained from a mosquito, (ii) the HB81P08 strain isolated from a human with neurological disease and (iii) a rodent isolate (ROD259466), as seen in Figure 4. Mice were inoculated with a 10^3^ pfu human, reference, or rodent strain by one of three routes: IC, IP and SC injections onto groups of ten 3-to-4-week-old individuals. The control mice were injected with PBS in duplicate groups using the same inoculation methods. Both the infected and uninfected control mice were monitored for up to 20 days with weighing sessions for individuals at two-day intervals or after observing significant events, such as mortality. The control experiments were realized later in time than the other ones.

Briefly, the percentage of the change in body weight was calculated based on the original weights (day-0). The average values for each virus on each day were then calculated for each method of inoculation. The survival curves were obtained in a similar way with the percentage of survival, based on the starting number of mice. The statistical results are available on Supplementary data 1.

Virus inoculated via the IC route had a significant impact on mouse growth, as seen in Figure 4. In fact, regardless of the inoculated strain, mice presented significantly lower weights than the controls (*p*-values ranged from 0.0084 to 0.07552). Symptomatic manifestations (tremors, apathy, and paralysis of posterior body) appeared four days post-infection (dpi) for one-third of the individuals infected with the strain ROD259466, against about 50% of the individuals infected with the other two viruses, as seen in Figure 4B,D,F. A mortality rate of one hundred percent was found between 8 and 10 days post-infection depending on the strain, with a trend toward shorter survival for the mosquito strain SAAR-1776, as seen in Figure 4A.

For IP or SC inoculations, mice inoculated with the human or rodent strains showed a growth rate greater or the same as the control individuals, as seen in Figure 4D,F. Only one individual exhibited symptoms at eight days post-infection with HB81P08 strain by IP route. This specimen is also the only one in his cohort to experience a fatal outcome, succumbing on day 10 post-infection. No other morbidity or mortality was observed in either IP or SC infection with ROD259466 or HB81P08 strain, as seen in Figure 4C–F. Otherwise, SAAR-1776-infected mice showed a significant slowdown in weight gain compared to the control group (*p*-value = 0.001505 for IP and *p*-value = 0.004571 for SC), with 60% apparent morbidity for the IP injected mice, a seen in Figure 4D,F, and 50% and 30% mortality nearly 15 days after IP and SC inoculations, respectively, as seen in Figure 4C,E.

## 4. Discussion

Here we report on the first isolation of USUV from two rodent species and a single species of shrew. It was shown that USUV is circulating among a wide variety of avian species in Africa [43,44] and Europe [45,46]. The detection of USUV RNA in bat brains was reported from Germany [9] while recent sero-survey studies showed that horses [47,48], dogs [49] and wild ruminants [50] are being increasingly exposed to USUV. Despite this growing number of direct or indirect viral detections, the existence of a non-avian reservoir remains unresolved. To the best of our knowledge, USUV has not been previously isolated from small mammals or vertebrates other than birds, bats, and humans [51].

Complete polyprotein sequences of the five new isolates are highly similar and were geographically isolated within 1 km of one another, as seen in Figure 1. Here, we found two unique substitutions in the capsid (C) protein shared by all five rodent and shrew strains, as seen in Figure 2. In addition to its role in flavivirus particle assembly [52], it has been shown that the C protein modulates host cell apoptosis [53,54]. As a consequence of the relative host diversity of arboviruses, structural proteins are subjected to select evolutionary pressures that can influence the host range [55,56]. The specific role of these two substitutions in USUV host-adaptation dynamics remains to be examined.

Despite these differences, a comparative study of in vivo USUV infection in mice showed that after IC inoculation, a randomly chosen rodent-derived strain shared the same features, in terms of lethality, with the mosquito-derived prototype and a human isolate, as seen in Figure 4. In fact, the different strains caused the death of all mice infected by this route. Almost no mortality was observed following IP or SC inoculations by rodent or human isolates of USUV, and infected mice presented a growth pattern greater or similar to the PBS-infected control group, as seen in Figure 4B,D,F. However, mortality or significant reductions in growth of SAAR-1776-infected mice were noted after both IP and SC inoculations, as seen in Figure 4C–F. Our results oppose the findings of Blázquez et al. [57] that describe no in vivo susceptibility to an IP SAAR-1776 challenge in mice. The number of cell passages or the mouse species as well as the age difference could explain this [58]: Blázquez et al. used eight-week-old Swiss mice [57] while we used four-week-old mice. Other unknown factors may eventually explain these differences. Mutations in envelope (E) and non-structural protein 3 (*NS3*) genes could also explain the phenotypic differences observed in mice for the prototype strain compared to rodent or human isolates. Furthermore, previous studies on the closely related flavivirus WNV showed that *E* and *NS3* genes are both involved in virus infectivity and neuroinvasiveness [59].

No disease manifestation or symptom indicative of a pathogenic infection was observed at the time of collection (no qualitative observation noting any tremors or reduction in mobility in captured small mammals). This suggests that rodents (*R. rattus* and *M. natalensis*) or shrews (*Crocidura* sp.) could be involved in the USUV replication cycle as an amplifying or reservoir host. The USUV-positive small mammals were trapped in Kedougou town, during a period in which *Culex quinquefasciatus* was the predominant mosquito species [60]. Considering that other related *Culex* species, such as *Culex neavei* and *Culex pipiens*, previously described as the USUV vector [61,62], and studies showing that *Culex quinquefasciatus* is an opportunistic feeder [63,64,65], a peridomestic transmission involving this mosquito species and small mammals probably occurred and could help explain our findings and previous detections of USUV in other mammals [49]. Further studies assessing USUV transmission in different mosquito species to and from rodents could improve our understanding of its enzootic cycles. As rodents and shrews are widely distributed in peridomestic habitats [26], contact with human populations often occurs with a potential risk of transmission, as observed with other pathogens [66].

A rodent reservoir may facilitate the spread and perpetuity of USUV, especially in Europe where dozens of rodent species coexist in different biomes and represent the most diverse group of mammals in the continent [67,68]. Our findings emphasize the need to consider the closer monitoring of small mammals in future active surveillance, public health, and epidemiological efforts, in responding to USUV in both Africa and Europe. 

## Figures and Tables

**Table 1:** Geographical positions of the five Usutu virus positive individuals.

GenBank Accession Number	Locality	Latitude Coordinates	Longitude Coordinates	ID Number	Host Species
MH727238	Kedougou	12.553997	−12.179781	ROD259466	*Mastomys natalensis*
MH727239	Kedougou	12.553614	−12.179837	ROD259496	*Mastomys natalensis*
MH727240	Kedougou	12.555794	−12.177485	CROC259520	Shrew *Crocidura* sp.
MH727241	Kedougou	12.555835	−12.178598	ROD259524	*Mastomys natalensis*
MH727242	Kedougou	12.55654	−12.177977	ROD259552	*Rattus rattus*

## Supplementary Materials

The following are available online at https://www.mdpi.com/1999-4915/11/2/181/s1, Table S1: Conventional RT-PCR systems used for arbovirus screening, Table S2: Number of small mammal specimens captured by species, Table S3: Phylogenetic inference of 28 complete polyprotein sequences, Supplementary Data 1: Statistical analysis for in vivo experiments.
