# Supplementary material for: Usutu Virus Isolated from Rodents in Senegal
Source: Viruses. 2019 Feb 21;11(2):181. doi: 10.3390/v11020181 (PMC6409855; doi:10.3390/v11020181)
Supplement: Supplementary file 1 [file viruses-11-00181-s001.pdf]

Table S1. Conventional RT-PCR systems used for arbovirus screening.

System detection	Primer sequences (5'-3')	References*
Pan-Alphavirus(nested PCR)	Alpha 1- : KYTCYTCIGTRTGYTTIGTICCIGG<br>Alpha 1+ : GAYGCITAYYTIGAYATGGTIGAIGG	Sanchez-Seco et al., 2001
	Alpha 2- : GCRAAIARIGCIGCIGCYTYIGGICC<br>Alpha 2+ : GIAAYTGYAAYGTIACICARATG	
Pan-Phlebovirus	Phlebo F1: TTTGCTTATCAAGGATTTGATGC<br>Phlebo F2: TTTGCTTATCAAGGATTTGACC<br>Phlebo R:TCAATCAGTCCAGCAAAGCTGGGATGCATCAT	Lambert and Lanciotti, 2009
Pan Flavivirus	FU1: TACAACATGATGGGVAARAGWGARAA<br>FD3: ARCATGTCTTCYGTBGTCATCCA	Kuno et al., 1998
Pan-Hantavirus- S segment	HTN-S4: GAIIGITGTCCACCAACATG<br>HTN-S6: AGCTCIGGATCCATITCATC	Arthur et al., 1993
Pan Hantavirus- L segment (nested PCR)	HAN-L-F1: 5′-ATGTAYGTBAGTGCWGATGC-3′	Klempa et al., 2006
	HAN-L-R1: 5′-AACCADTCWGTYCCRTCATC-3′	
	HAN-L-F2: 5′-TGCWGATGCHACIAARTGGTC-3′<br>HAN-L-R2: 5′-GCRTCRTCWGARTGRTGDGCAA-3′	

*Sánchez-Seco MP, Rosario D, Quiroz E, Guzmán G, Tenorio A. A generic nested-RT-PCR followed by sequencing for detection and identification of members of the alphavirus genus. J Virol Methods. 2001 Jun; 95(1-2):153-61.

*Lambert AJ, Lanciotti RS. Consensus amplification and novel multiplex sequencing method for S segment species identification of 47 viruses of the Orthobunyavirus, Phlebovirus, and Nairovirus genera of the family Bunyaviridae. J ClinMicrobiol. 2009 Aug;47(8):2398-404. doi: 10.1128/JCM.00182-09. Epub 2009 Jun 17.

*Kuno G, Chang G-J, Tsuchiya K R, Karabatsos N, Cropp C B. Phylogeny of the genus *Flavivirus*. J Virol. 1998;72:73–83.

*Arthur RR, Lofts RS, Gomez J, Glass GE, Leduc JW, Childs JE. Grouping of Hantaviruses by small (S) genome segment polymerase chain reaction and amplification of viral RNA from wild-caught rats. Am J Trop Med Hyg 1993 Mar;48(3).

*Klempa B, Fichet-Calvet E, Lecompte E, et al. Hantavirus in African Wood Mouse, Guinea. EmergingInfectiousDiseases. 2006;12(5):838-840. doi:10.3201/eid1205.051487.

Table S2. Number of small mammal specimens captured by species.

Small mammal species	Number of captured specimens
Black rat *Rattus rattus*	403
Shrew*Crocidura sp*	296
Mus musculus	247
Mastomys natalensis	241
Mastomys erythroleucus	94
Arvicanthis niloticus	79
Praomys daltoni	52
Cricetomys gambianus	1
Gerbilliscus gambianus	1
Total	1414

Table S3. Phylogenetic inference of 28 complete polyprotein sequences.

Accession number	Strain/Isolate	Host	2-letter ISO country code	Collection year
KF573410	MB119/06	Mosquito	ES	2006
KJ438774	V248	Avian species	DE	2012
KJ438771	V312	Avian species	DE	2013
KY426770	V575	Avian species	DE	2016
KY426769	V574	Avian species	DE	2016
KY426768	V541	Avian species	DE	2016
KY426767	V540	Avian species	DE	2016
KY426766	V528	Avian species	DE	2016
KY426765	V507	Avian species	DE	2016
KY426764	V504	Avian species	DE	2016
KY426763	V501	Avian species	DE	2016
KY426762	V500	Avian species	DE	2016
KY426761	V499	Avian species	DE	2016
KY426760	V494	Avian species	DE	2016
KY426759	V492	Avian species	DE	2016
KY426758	V491	Avian species	DE	2016
KY426757	V490	Avian species	DE	2016
KY426756	V489	Avian species	DE	2016
KY426755	V472	Avian species	DE	2016
KY426754	V466	Avian species	DE	2015
KY426753	V465	Avian species	DE	2015
KY426752	V464	Avian species	DE	2015
KY426751	V461	Avian species	DE	2015
KY426750	V453	Avian species	DE	2015
KY263626	Lincent	Avian species	BE	2016
KY263625	Frameries	Avian species	BE	2016
KY263624	Flemalle	Avian species	BE	2016
KY199558	VD54359	Avian species	DE	2016
KY199557	65436	Avian species	DE	2016
KY199556	65396	Avian species	DE	2016
KY315178	U1609393	Avian species	BE	2016
KX601691	HautRhin7316	Avian species	FR	2015
KX601690	HautRhin7315	Avian species	FR	2015
KY128482	6004	Avian species	NL	2016
KU664608	cloneME	Avian species	DE	2015
KJ438781	V86	Avian species	DE	2011
KJ438768	6950	Mosquito	DE	2013
KJ438767	2794	Mosquito	DE	2011
KJ438705	1477	Mosquito	DE	2010
KM659877	Bonn	Avian species	DE	2014
KJ859683	BAT2	Bat	DE	2013
KJ438737	4356	Mosquito	DE	2011
KC754958	ArB1803	Mosquito	CF	1969
KC754957	ArD192495	Mosquito	SN	2007
KC754956	ArD101291	Mosquito	SN	1993
KC754955	HB81P08	Human	CF	1981
KC754954	ArD19848	Mosquito	SN	1974
JF266698	Italia2009	Avian species	IT	2009
EF206350	Budapest	Avian species	HU	2005
KJ438779	MeiseH	Avian species	AT	2002
KJ438739	V391	Avian species	DE	2013
KJ438736	6424	Mosquito	DE	2012
AY453412	SAAR-1776	Mosquito	ZA	1959

AY453411	Vienna2001	Avian species	AT	2001
HM569263	Bologna2009	Human	IT	2009
KJ859682	BAT1	Bat	DE	2013
MH727238	ROD259466	Rodent	SN	2013
MH727239	ROD259496	Rodent	SN	2013
MH727240	CROC259520	Rodent	SN	2013
MH727241	ROD259524	Rodent	SN	2013
MH727242	ROD259552	Rodent	SN	2013

Supplementary data 1: Statistical analysis for in vivo experiments
HB = Human strain HBP08
RB = Rodent strain IP259466
SAAR = Reference strain SAAR-1776
ns = Non significant
IC = Intracerebral infection
IP = Intraperitoneal infection
SC = Subcutaneous infection

Weight Figures

```
#IC.w
wilcox.test(SAAR.ic$Body.weight...change.mean, HB.ic$Body.weight...change.mean) #ns
wilcox.test(SAAR.ic$Body.weight...change.mean, R25.ic$Body.weight...change.mean) #ns
wilcox.test(SAAR.ic$Body.weight...change.mean, control.ic$Body.weight...change.mean) # p-value = 0.07552
wilcox.test(HB.ic$Body.weight...change.mean, R25.ic$Body.weight...change.mean) #ns
wilcox.test(HB.ic$Body.weight...change.mean, control.ic$Body.weight...change.mean) # p-value = 0.07549
wilcox.test(R25.ic$Body.weight...change.mean, control.ic$Body.weight...change.mean) # p-value = 0.0084

#IP.w
wilcox.test(SAAR.ip$Body.weight...change.mean, HB.ip$Body.weight...change.mean) #p-value = 0.001505
wilcox.test(SAAR.ip$Body.weight...change.mean, R25.ip$Body.weight...change.mean) # p-value = 0.005196
wilcox.test(SAAR.ip$Body.weight...change.mean, control.ip$Body.weight...change.mean) #p-value = 0.00105
wilcox.test(HB.ip$Body.weight...change.mean, R25.ip$Body.weight...change.mean) #ns
wilcox.test(HB.ip$Body.weight...change.mean, control.ip$Body.weight...change.mean) # p-value = 0.008931
wilcox.test(R25.ip$Body.weight...change.mean, control.ip$Body.weight...change.mean) # ns

#SC.w
wilcox.test(SAAR.sc$Body.weight...change.mean, HB.sc$Body.weight...change.mean) #p-value = 0.004556
wilcox.test(SAAR.sc$Body.weight...change.mean, R25.sc$Body.weight...change.mean) # p-value = 0.01398
wilcox.test(SAAR.sc$Body.weight...change.mean, control.sc$Body.weight...change.mean) #ns
wilcox.test(HB.sc$Body.weight...change.mean, R25.sc$Body.weight...change.mean) #ns
wilcox.test(HB.sc$Body.weight...change.mean, control.sc$Body.weight...change.mean) # p-value=0.004556
wilcox.test(R25.sc$Body.weight...change.mean, control.sc$Body.weight...change.mean) # p-value=0.03756
```

Survival Figures

```
#IC.s
wilcox.test(SAAR.ic.s$Percent_survival, HB.ic.s$Percent_survival) #ns
wilcox.test(SAAR.ic.s$Percent_survival, R25.ic.s$Percent_survival) #ns
wilcox.test(HB.ic.s$Percent_survival, R25.ic.s$Percent_survival) #ns

#IP.s
wilcox.test(SAAR.ip.s$Percent_survival, HB.ip.s$Percent_survival) #p-value = 0.00783
wilcox.test(SAAR.ip.s$Percent_survival, R25.ip.s$Percent_survival) #p-value = 7.84e-05
wilcox.test(HB.ip.s$Percent_survival, R25.ip.s$Percent_survival) #p-value = 0.003255

#SC.s
```

```
wilcox.test(SAAR.sc.s$Percent_survival, HB.sc.s$Percent_survival) #p-value = 2.261e-05
wilcox.test(SAAR.sc.s$Percent_survival, R25.sc.s$Percent_survival) #p-value = 2.261e-05
wilcox.test(HB.sc.s$Percent_survival, R25.sc.s$Percent_survival) #ns
```
